# Primary clear cell renal carcinoma cells display minimal mitochondrial respiratory capacity resulting in pronounced sensitivity to glycolytic inhibition by 3-Bromopyruvate

**DOI:** 10.1038/cddis.2014.545

**Published:** 2015-01-08

**Authors:** H Nilsson, D Lindgren, A Mandahl Forsberg, H Mulder, H Axelson, M E Johansson

**Affiliations:** 1 Department of Laboratory Medicine Malmö, Center for Molecular Pathology, Lund University, Skåne University Hospital, Malmö, Sweden; 2 Department of Laboratory Medicine Lund, Division of Translational Cancer Research, Lund University, Lund, Sweden; 3 Department of Urology, Clinical Sciences Malmö, Lund University, Malmö, Sweden; 4 Department of Clinical Sciences, Unit of Molecular Metabolism, Lund University Diabetes Centre, Malmö, Sweden

## Abstract

Changes of cellular metabolism are an integral property of the malignant potential of most cancer cells. Already in the 1930s, Otto Warburg observed that tumor cells preferably utilize glycolysis and lactate fermentation for energy production, rather than the mitochondrial oxidative phosphorylation dominating in normal cells, a phenomenon today known as the Warburg effect. Even though many tumor types display a high degree of aerobic glycolysis, they still retain the activity of other energy-producing metabolic pathways. One exception seems to be the clear cell variant of renal cell carcinoma, ccRCC, where the activity of most other pathways than that of glycolysis has been shown to be reduced. This makes ccRCC a promising candidate for the use of glycolytic inhibitors in treatment of the disease. However, few studies have so far addressed this issue. In this report, we show a strikingly reduced mitochondrial respiratory capacity of primary human ccRCC cells, resulting in enhanced sensitivity to glycolytic inhibition by 3-Bromopyruvate (3BrPA). This effect was largely absent in established ccRCC cell lines, a finding that highlights the importance of using biologically relevant models in the search for new candidate cancer therapies. 3BrPA markedly reduced ATP production in primary ccRCC cells, followed by cell death. Our data suggest that glycolytic inhibitors such as 3BrPA, that has been shown to be well tolerated *in vivo*, should be further analyzed for the possible development of selective treatment strategies for patients with ccRCC.

World wide, ~340 000 patients are diagnosed with renal cell carcinoma (RCC) annually.^1^ The majority (75%) is of the clear cell subtype, ccRCC.^2^ A problematic therapeutic feature of ccRCC is that if curative surgery fails, traditional radio- or chemotherapy have very limited effect.^3^ Despite recent advances using targeted anti-angiogenic therapies, the 5-year survival for patients with metastatic disease is still only a dismal 10%. Increased understanding of this disease that could result in development of new therapies is therefore of great importance.

ccRCC derives its name from the histologically empty (clear) appearance of the cancer cells, caused by large deposits of glycogen and lipid droplets in the cytoplasm.^4^ The initiating genetic event seems to be loss of function of the tumor suppressor protein von Hippel Lindau, pVHL, found in over 90% of all cases.^5, 6^ This results in aberrant activation of the hypoxia inducible factors HIF-1*α* and HIF-2*α*, transcription factors normally active only at hypoxia, controlling the cellular adaptation to oxygen shortage, such as induction of angiogenesis, cell cycle arrest and pro-survival factors.^7, 8^ The resulting pseudohypoxic phenotype in ccRCC tumors leads to massive angiogenesis, as well as an extensive metabolic reprogramming, including a shift from mitochondrial oxidative phosphorylation to non-oxygen-requiring glycolysis. In normal cells, when oxygen is abundant, glycolysis is used to metabolize glucose into pyruvate, which enters the citric acid cycle in the mitochondria. ATP is subsequently produced by oxidative phosphorylation, driven by the reducing equivalents formed in the citric acid cycle. During hypoxic conditions, pyruvate is instead fermented to lactate and excreted from the cell. Already in the 1930s the German physiologist Otto Warburg described that tumor cells preferentially utilize the glycolytic pathway to produce lactate also when oxygen is available, a phenomenon today known as the Warburg effect. Although such aerobic glycolysis yields less ATP per molecule of glucose than its full oxidation to CO_2_ and H_2_O in mitochondrial metabolism, this pathway provides tumor cells with building blocks needed for growth and proliferation. The current view is that even though increased aerobic glycolysis is one of the characteristics of tumor cells, mitochondrial and other metabolic pathways are usually still maintained alongside with a high glycolytic rate in most cancer forms.^9^

Recent studies indicate that the metabolism of ccRCC tumors differs from other cancer forms in that most pathways besides glycolysis are downregulated.^10, 11, 12, 13^ In this report, we show that the number and respiratory capacity of mitochondria are strongly reduced in ccRCC cells, which instead exhibit high glycolytic activity. We hypothesized that this renders ccRCC cells highly sensitive to inhibition of glycolysis. To test this hypothesis, we exposed primary ccRCC cells to 3-bromopyruvate (3BrPA), a pyruvate analog primarily described as a glycolytic inhibitor. We show that primary human ccRCC cells, in contrast to normal kidney cells and also established ccRCC cell lines, were highly sensitive to inhibition by 3BrPA. The possibility to utilize metabolic inhibitors might be a promising therapeutic alternative to specifically target ccRCC tumors that should be further studied.

## Results

### ccRCC tumors contain few mitochondria and are highly glycolytic

**Figure 1:**
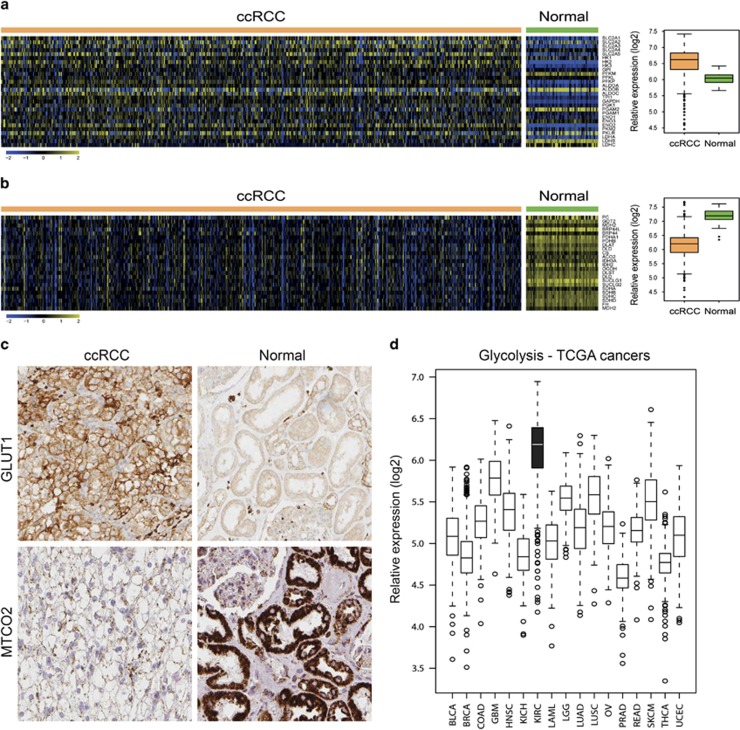
ccRCC cells have low mitochondrial content and are highly glycolytic. Heatmaps illustrating relative mRNA transcript levels of genes involved in the glycolytic pathway (**a**) or mitochondrial metabolism (**b**) in 505 ccRCC tumor and 70 normal kidney samples as obtained from the TCGA database. Boxplots summarize the gene expression data presented in the heatmaps. For each sample the mean expression values of the glycolytic and mitochondrial genes, respectively, was calculated. These mean values were then plotted stratified on ccRCCs and normal kidney samples. (**c**) Immunohistochemical staining of ccRCC or normal kidney tissue samples for Glut1 (upper panel) or MTCO2 (lower panel). (**d**) Box plot showing the relative expression levels of glycolytic genes in various cancer types included in the TCGA data collection. BLCA, Bladder Urothelial Carcinoma; BRCA, Breast invasive carcinoma; COAD, Colon adenocarcinoma; GBM, Glioblastoma multiforme; HNSC, Head and Neck squamous cell carcinoma; KICH, Kidney Chromophobe; KIRC, Kidney renal clear cell carcinoma; LAML, Acute Myeloid Leukemia; LGG, Brain Lower Grade Glioma; LUAD, Lung adenocarcinoma; LUSC, Lung squamous cell carcinoma; OV, Serous Cystadenocarcinoma; PRAD, Prostate adenocarcinoma; READ, Rectum adenocarcinoma; SKCM, Stomach adenocarcinoma; THCA, Thyroid Carcinoma; UCEC, Uterine Corpus Endometrial Carcinoma

**Figure 2:**
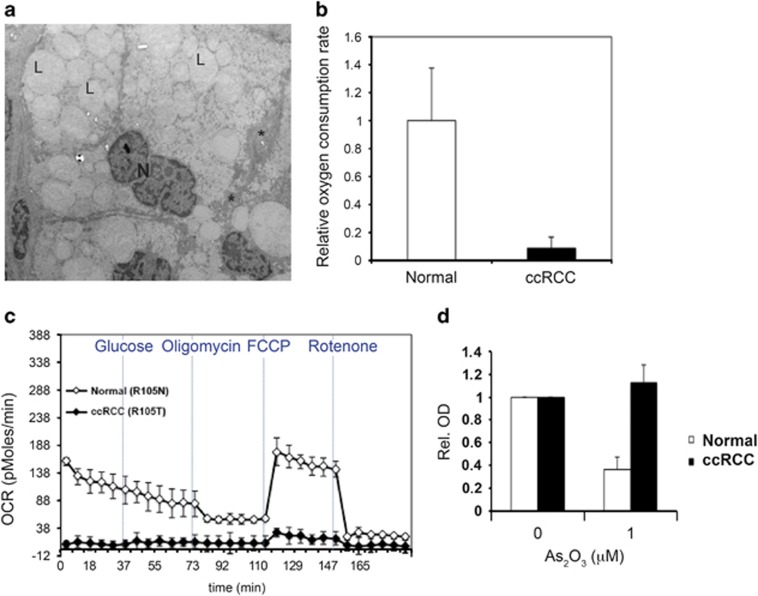
Primary ccRCC cells show little sensitivity to respiratory inhibition. (**a**) Electron microscope image from a ccRCC tissue sample. Note the large amount of lipid droplets (L) and glycogen deposits (*) in the cytoplasm, and also the apparent absence of mitochondria. *N*=nucleus. (**b**) The mean values of the relative basal OCR in cultured primary ccRCC cells (R103T, R105T, R110T, R130T and R138T) compared with normal kidney epithelial cells (R103N, R104N and R105N). OCR was determined using the Seahorse technique, data were normalized to number of cells. (**c**) Seahorse experiment showing the low maximal respiratory capacity after addition of FCCP in the same amount of cells of the primary R105T ccRCC culture compared with the R105N normal kidney epithelial cells. Note the very low basal OCR level of the primary ccRCC cells. (**d)** WST-1 assay showing the high tolerance of primary ccRCC cells to inhibition of mitochondrial respiration by treatment with As_2_O_3_ for 3 days, as compared with normal kidney epithelial cells. Data represent the mean values from two different normal and ccRCC samples (R104N, R117N and R111T, R120T, respectively)

To validate the metabolic characteristics of ccRCC cells described above, we analyzed the expression of genes involved in the glycolytic pathway in ccRCC tumor and normal kidney samples included in The Cancer Genome Atlas (TCGA) data set. As shown in Figure 1a, the majority of analyzed glycolytic genes were expressed at elevated levels in ccRCC compared with matched normal samples. This was confirmed at the protein level, as exemplified by immunohistochemical stainings of GLUT1 in ccRCC and normal kidney tissue samples (Figure 1c, upper panel). The expression levels of the glycolytic genes were also confirmed to be clearly elevated in ccRCC tumors compared with other cancers included in the TCGA data collection, as illustrated in Figure 1d. In contrast, genes involved in mitochondrial regulation and oxidative phosphorylation were downregulated in ccRCC samples compared with normal kidney samples (Figure 1b). We next analyzed the mitochondrial content in ccRCC tumors. Staining of ccRCC and normal kidney tissue for mitochondrial encoded cytochrome *c* oxidase II (MTCO2) revealed high expression in normal epithelial cells of the proximal tubules, whereas the expression in ccRCC cells was strongly reduced (Figure 1c, lower panel). The low mitochondrial load in ccRCC samples was further substantiated using electron microscopy. Images of ccRCC tissue clearly demonstrated the characteristic accumulation of cytoplasmic lipid droplets and glycogen deposits, but also that very few, if any, mitochondria could be detected (Figure 2a). With purpose to obtain an *in vitro* model allowing for further characterization of the metabolic features of ccRCC cells, tumor as well as normal primary proximal tubular cells from patients diagnosed with ccRCC were isolated and cultured. As a quantification of the mitochondrial load in cultured ccRCC cells, the ratio between mitochondrial and nuclear DNA was determined with quantitative PCR analysis. In Supplementary Figure 1A, the reduction in mitochondrial DNA content of primary ccRCC cells compared with normal kidney cells is clearly demonstrated.

These data urged us to analyze the capacity of ccRCC cells to utilize mitochondrial oxidative phosphorylation for energy production. Measurement of cellular oxygen consumption rate (OCR) by the Seahorse technique can be used to quantify mitochondrial respiration. As shown in Figure 2b, the basal OCR per cell was up to 10 times higher in normal cells compared with ccRCC tumor cells, indicating a very low usage of oxidative phosphorylation as energy source in ccRCC cells. Addition of oligomycin, an inhibitor of the ATP synthase, reduced OCR in normal cells, as expected. However, the already very low basal OCR levels of the ccRCC cells prohibited us from detecting any further reduction in response to oligomycin treatment in these cells. The uncoupler FCCP disrupts the mitochondrial proton gradient driving ATP production, giving a measurement of maximal respiratory capacity. OCR measurements after addition of FCCP showed a slight increase in oxygen consumption in the primary ccRCC cells, indicating that some respiratory activity was present in these cells; however, compared with the primary normal samples the ccRCC cells displayed a remarkably low respiratory capacity (Figure 2c).

The low mitochondrial respiratory rate of primary ccRCC cells was further illustrated by treatment with As_2_O_3_, which inhibits mitochondrial respiration. As_2_O_3_ had negligible effect on primary ccRCC cells, while normal primary kidney epithelial cells did not tolerate this treatment (Figure 2d). Similarly, treatment with the mitochondrial complex I-inhibitor metformin at concentrations reported to induce cell death in several cancer cell lines^14, 15, 16^ had no effect on viability of primary ccRCC cells (Supplementary Figure 1B). Together, these results confirm that ccRCC cells do not rely on oxidative phosphorylation for ATP production, instead indicating a critical role for glycolysis in their energy metabolism.

### 3BrPA inhibits the growth of primary ccRCC cells

**Figure 3:**
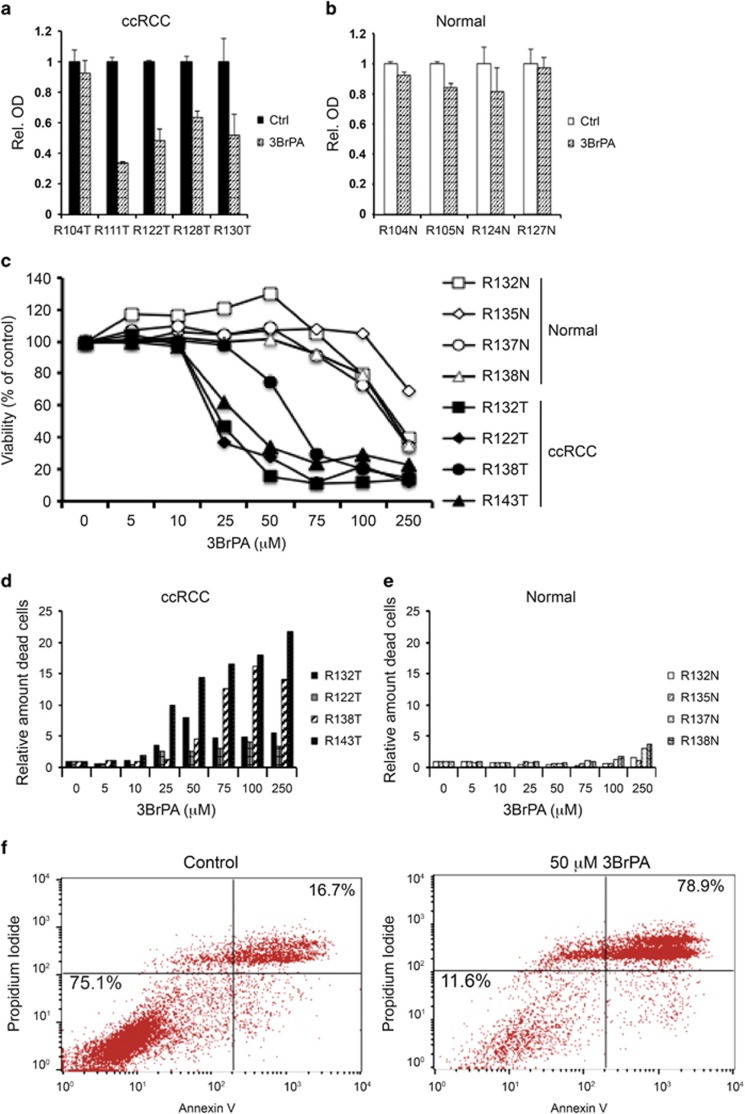
3BrPA inhibits growth of primary ccRCC cells. WST-1 assay showing the effect of 50 μM 3BrPA on five different cultures of primary ccRCC (**a**) and four cultures of normal kidney cells (**b**), after 3 days of treatment. (**c**) Viability determined by nucleocounter of primary cultured cells from four normal or ccRCC samples after incubation for 24 h with increasing concentration of 3BrPA. (**d** and **e**) Relative number of dead cells determined by nucleocounter after 24 h treatment with the indicated concentrations of 3BrPA in ccRCC (**d**) or normal (**e**) cells. (**f)** Percentage apoptotic (Annexin V and Propidium Iodide-positive) ccRCC cells in control (left) or after 24 h treatment with 50 *μ*M 3BrPA (right). Results from one representative experiment are shown

The low mitochondrial capacity and high glycolytic profile of ccRCC cells suggest inhibition of glycolysis as a potentially effective method to limit energy supply in these cells. To test this hypothesis, we chose to use the glycolytic inhibitor 3BrPA (Supplementary Figure 2A). The effect of 3BrPA was first studied using WST-1 assay. As shown in Figure 3a, three out of four tested tumors were sensitive to 50 *μ*M 3BrPA, whereas all four tested normal cultures were largely unaffected after 3 days of treatment (Figure 3b). Following treatment for 6 days, the effect was even stronger in the tumor cells. Again, this treatment period was also well tolerated by the normal kidney cells (Supplementary Figure 2B and C). Interestingly, cells from the only primary ccRCC culture that did not respond to 3BrPA treatment (R104T) showed an elevated basal OCR and increased maximal respiratory capacity in the Seahorse assay, suggesting retained mitochondrial capacity and less dependence on glycolysis in this specific tumor (Supplementary Figure 3).

The colorimetric WST-1 assay is based on the reduction of a tetrazolium salt into a formazan dye by the succinate-tetrazolium reductase system in the respiratory chain of mitochondria. The very low mitochondrial content of the ccRCC tumor cells might thus interfere with the results. We therefore shifted to counting the number of dead and live cells using the viability and cell count assay on the Nucleocounter 3000. The viability of ccRCC samples compared with normal samples after 24 h treatment with increasing concentrations of 3BrPA is shown in Figure 3c. Tumor cell viability was decreased already at 25 *μ*M 3BrPA, whereas normal cells tolerated significantly higher concentrations. The IC_50_ values of each of these cultures are presented in Supplementary Table 1. In Figures 3d and e the relative number of dead cells from the same experiments are shown. Again, the dramatic difference in the toxicity of 3BrPA towards ccRCC tumor cells compared with normal kidney cells is clearly seen. The amount of apoptotic cells after 3BrPA treatment was also determined by flow cytometry analysis of Annexin V/Propidium Iodide-stained cells. Figure 3f shows the increase in double-positive apoptotic primary ccRCC cells after 24-h treatment with 50 μM 3BrPA.

### 3BrPA inhibits lactate excretion and ATP production in ccRCC cells

**Figure 4:**
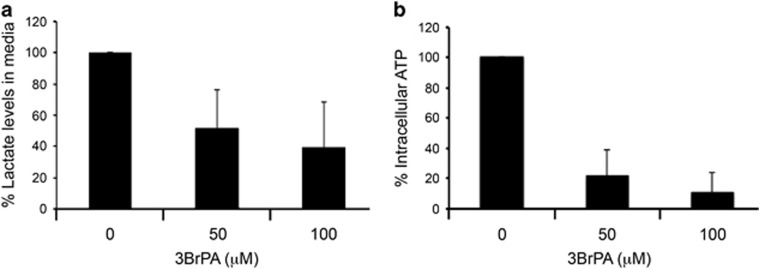
3BrPA inhibits lactate excretion and ATP production in primary ccRCC cells. (**a**) Lactate levels in medium from primary ccRCC cultures were measured after 1-h treatment with 50 or 100 *μ*M 3BrPA. Data presented as relative lactate levels compared with medium from untreated cells. Data shown are an average of results from nine different tumor samples (R110T, R111T, R122T, R132T, R133T, R135T, R138T, R139T and R140T). (**b**) Intracellular ATP levels in primary ccRCC cells were analyzed by a luciferase assay after 1-h treatment with 50 or 100 μM 3BrPA. Data shown are an average of results from 10 different tumor samples (R110T, R111T, R122T, R130T, R132T, R133T, R135T, R138T, R139T and R140T)

3BrPA has been described as an inhibitor of glycolysis, by blocking the activity of hexokinase or glyceraldehyde dehydrogenase.^17, 18^ In the final step of aerobic glycolysis, pyruvate is converted into lactate; thus measurement of lactate excretion can be used as an estimate of aerobic glycolytic flux. In primary ccRCC cells, addition of 3BrPA resulted in a significant reduction in the amount of lactate in media within 1 h (Figure 4a). The same treatment also resulted in a dramatic decrease in intracellular ATP levels (Figure 4b). These results suggest that 3BrPA inhibits glycolysis in primary ccRCC cells, resulting in the depletion of ATP levels.

### Established renal carcinoma cell lines are less sensitive to 3BrPA treatment

**Figure 5:**
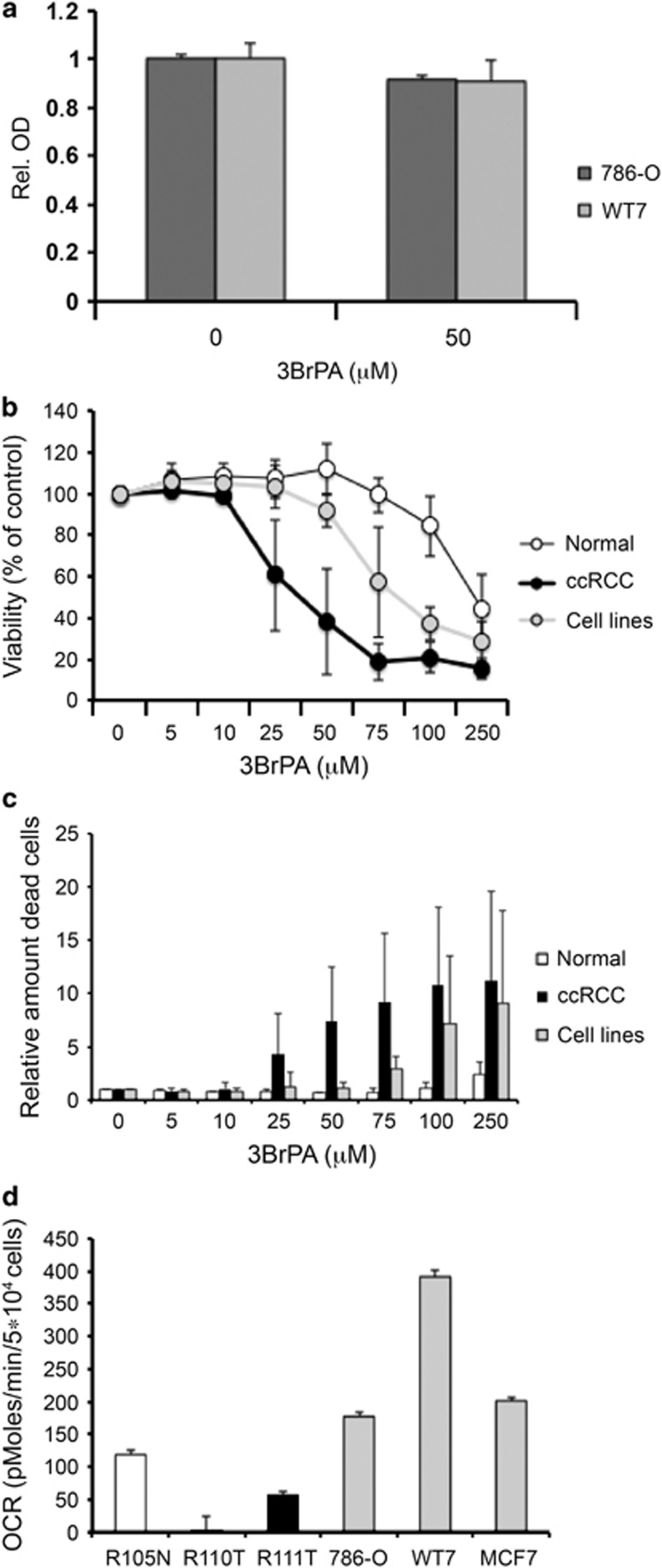
Established cell lines are less sensitive to glycolytic inhibition. (**a**) Effect on growth of the established ccRCC cell lines 786-O and WT7 after treatment with 50 *μ*M 3BrPA for 24 h as determined by WST-1 assay. (**b**) Dose–response curve showing the average viability of seven established ccRCC cell lines (786-O, WT7, SKRC7, SKRC10, SKRC17, SKRC21 and SKRC52) compared with average from four cultured primary normal samples (R132N, R135N, R137N and R138N) or ccRCC cells (R132T, R122T, R138T and R143T) after 24-h treatment with increasing concentrations of 3BrPA, determined using the Nucleocounter. (**c**) Relative amount of dead cells from the same experiment as in **b**, after 24-h treatment with indicated concentrations of 3BrPA. (**d**) Basal OCR per cell measured by Seahorse in one normal kidney sample, two different primary ccRCC samples, two established ccRCC cell lines (786-O, WT-7) and one breast cancer cell line (MCF7)

Established ccRCC cell lines are regularly used for *in vitro* studies of ccRCC. Therefore, we analyzed the effects of 3BrPA in the established ccRCC cell lines 786-O and WT7. Intriguingly, the viability of these cells was not affected by treatment with 50 μM 3BrPA (Figure 5a). The 786-O cell line is established from a ccRCC tumor that lacks functional VHL, and WT7 is a subclone of this cell line where VHL has been reintroduced. Both these cell lines lack HIF-1*α* expression.^19^ Five additional ccRCC cell lines (SKRC7, SKRC10, SKRC17, SKRC21 and SKRC52), commonly used to study this tumor type *in vitro*, were included in these studies, in order to determine whether also other ccRCC cell lines were equally resistant to 3BrPA, and also to ensure that the lack of HIF-1α was not a determining factor in the resistance of the established cell lines to 3BrPA treatment. All tested continuous ccRCC cell lines showed an increased resistance to glycolytic inhibition by 3BrPA treatment compared with primary ccRCC cells, although they appeared somewhat more sensitive than primary normal kidney cells (Figures 5b and c). The individual IC_50_ values of each cell line are presented in Supplementary Table 1. Cell cycle analysis of 3BrPA-treated cells revealed no major effects in primary normal or ccRCC cells, whereas the established cell lines showed a tendency towards S-phase arrest after treatment with 50 *μ*M 3BrPA (Supplementary Figure 4).

Notably, 786-O and WT-7 cells displayed an increased basal OCR relative to primary ccRCC cells (Figure 5d), and the maximal respiratory capacity was also higher in these cells. As a comparison, the basal OCR of the breast cancer cell line MCF7 is also shown. In line with these findings, quantifications of the mitochondrial DNA content indicated a higher mitochondrial load in the established cell lines compared with primary ccRCC cells (Supplementary Figure 1). These results suggest that established cell lines are less dependent on glycolysis than primary ccRCC cells, and thus less sensitive to glycolytic inhibition by 3BrPA.

### MCT1 is highly expressed in ccRCC cells

**Figure 6:**
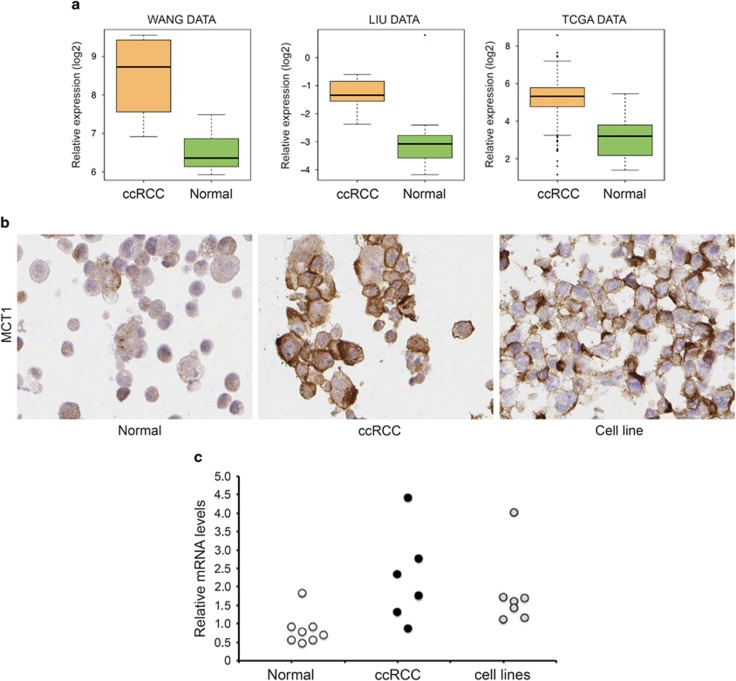
SLC16A1 expression is elevated in ccRCC cells. (**a**) RNA levels of the monocarboxylate transporter *SLC16A1* are elevated in ccRCC tissue compared with normal kidney. Data obtained from TCGA data collection and two separate publically available gene arrays (see Materials and Methods). (**b**) Immunohistochemical staining of MCT1 in cultured primary ccRCC, normal kidney epithelial cells and the continuous cell line SKRC17. The images shown are representative of four separate cultures of each cell type that were stained for MCT1. (**c**) Relative *SLC16A1* mRNA levels in eight normal kidney cultures (R104N, R117N, R124N, R127N, R130N, R131N, R132N and R134N), six ccRCC samples (R103T, 3122T, R125T, R130T, R132T and R173T) and the seven established cell lines, determined by quantitative real-time PCR

Being a pyruvate analogue, 3BrPA has been reported to be transported into cells by the monocarboxylate transporter 1 (MCT1 or *SLC16A1*),^20^ a proton-linked pump that catalyzes the transport of monocarboxylates such as lactate and pyruvate across the cell membrane. The expression of this pump is induced in hypoxic and glycolytic cells. Data obtained from publically available gene expression data sets, together with the TCGA, showed that ccRCC tumors express *SLC16A1* at significantly higher levels than normal kidney cells (Figure 6a). These data were confirmed at the protein level in cultured primary ccRCC and normal kidney cells (Figure 6b). The increased resistance of the established ccRCC cell lines to 3BrPA treatment raised the question regarding the expression of *SLC16A1* in these cells. As exemplified in Figure 6b, protein levels of MCT1 in the SKRC17 cell line were almost as high as in the primary ccRCC cells. Similar results were obtained also from the other cell lines. Indeed, quantification of the relative expression levels of *SLC16A1* mRNA in these different cell types confirmed a pattern where primary normal cells displayed low expression levels of *SLC16A1*, whereas both the primary ccRCC cells and the established cell lines expressed increased levels of this transporter (Figure 6c).

## Discussion

In ccRCC cells, the pseudohypoxic state following HIF activation due to loss of VHL results in a shift from mitochondrial to glycolytic metabolism.^21^ Recently, a significant difference in the metabolic profile of ccRCC compared with other investigated tumor types was described.^10^ Even though the Warburg effect is seen in many cancer forms, ccRCC seems to be uniquely dependent on glycolysis, showing a reduction of most other energy-generating metabolic processes. Investigating the functional basis for this shift, we studied ccRCC tissue by electron microscopy and immunohistochemistry, finding a severe reduction in the number of mitochondria. Despite using a protocol developed for mitochondrial visualization,^22^ few if any mitochondria could be detected in the cytoplasm of ccRCC cells. This was reflected in primary ccRCC cells in culture by a very low basal OCR as measured by the Seahorse technique, low mitochondrial DNA content and a correspondingly high tolerance to inhibitors of mitochondrial respiration at concentrations shown to be lethal to other tumor cells in culture.^14, 15, 16^ The low mitochondrial content results in a limited ability of primary ccRCC cells to increase OCR and flux through the electron transport chain after uncoupling of the mitochondrial ATPase, illustrated by addition of the uncoupler FCCP in the Seahorse assay. ccRCC cells thus seem to be unable to switch to oxidative phosphorylation for energy production, implying a low risk for development of resistance upon glycolytic inhibition.

Constitutive HIF signaling also renders ccRCC tumors exceptionally well vascularized. The capillary network supplies tumor cells well with oxygen and nutrients, which might explain how they can rely almost solely on glycolysis. This renders ccRCC cells more vulnerable to anti-angiogenic therapy,^23^ and as we hypothesize, to inhibition of the glycolytic pathway.

The latter aspect was tested by exposing primary ccRCC cells to the glycolytic inhibitor 3BrPA, a compound shown to be well tolerated *in vivo*,^24, 25^ recently approved for phase I clinical trials in metastatic liver cancer. We found that primary ccRCC cells indeed were sensitive to 3BrPA at concentrations tolerated by normal primary kidney cells. Addition of 3BrPA to primary ccRCC cells resulted in reduced lactate excretion and a drastic drop in ATP production, suggesting that 3BrPA treatment induced cell death due to energy depletion following inhibition of the glycolytic pathway.

The inhibitory effect of 3BrPA on glycolysis has both been described to function through targeting of glyceraldehyde dehydrogenase as well as of the rate-limiting enzyme hexokinase, catalyzing the conversion of glucose to glucose-6-phosphate in the first step of the glycolytic reaction.^17, 18^ Interestingly, recent studies suggest that hexokinase, highly expressed in ccRCC tumor cells, has an oncogenic function in these cells,^26^ further arguing for the benefit of using 3BrPA as a therapeutic agent in ccRCC.

Although 3BrPA has mainly been described as an inhibitor of glycolysis, it has also been reported to target mitochondrial function by inhibiting mitochondrially bound hexokinase^17, 27^ or by inhibiting the activity of succinate dehydrogenase (complex II in the respiratory chain).^28, 29^ These effects seem to vary depending on concentration, cell type and on the carbon source available for energy metabolism, where the greatest inhibitory effect of 3BrPA on mitochondrial function was found when glucose supply was limited.^28^ However, in the case of ccRCC cells, their nutrient-rich environment and low mitochondrial content implies that the toxic effect of 3BrPA on these cells most likely acts through glycolytic inhibition. In further support of this theory is our results demonstrating a high tolerance of primary ccRCC cells to known inhibitors of the respiratory chain, as well as the reduction in lactate excretion following 3BrPA treatment.

Uptake of 3BrPA has been reported to be dependent on the monocarboxylate transporter MCT1, or *SLC16A1*. The expression of this lactate transporter correlates to a more pronounced glycolytic profile.^20^ In this study we showed that the levels of *SLC16A1* was elevated both in ccRCC tissue samples and in cultured primary ccRCC cells compared with normal kidney cells, suggesting an increased uptake of 3BrPA specifically in ccRCC tumor cells. The low *SLC16A1* levels found in primary normal kidney cells probably limit their uptake of 3BrPA. Although this may add a level of protection towards any off-target effects of high-dose 3BrPA treatment, we do not believe that the low *SLC16A1* levels is the main explanation for the increased resistance of the normal cells to 3BrPA. One of the normal kidney cell samples (R124N) displayed elevated expression of *SLC16A1* compared with the others, yet these cells did not show an increased sensitivity to 3BrPA treatment, arguing that the absolute levels of *SLC16A1* is not the critical factor determining sensitivity to 3BrPA. Instead, we hypothesize that the cellular metabolic feature is a crucial aspect, where the constrained glycolytic profile of ccRCC cells renders them highly sensitive, whereas normal cells with a broader metabolic repertoire will be spared.

We additionally found that established ccRCC cell lines also were less sensitive to 3BrPA treatment, despite the presence of *SLC16A1* at almost the same elevated levels as in the primary ccRCC cells. No correlation between 3BrPA IC_50_ values and SLC16A1 expression levels was found among the continuous cell lines. Expression levels of *SLC16A1* in the SKRC10 cell line was significantly higher compared with the other established cell lines. Again, this was not reflected in any difference in the sensitivity of these cells to 3BrPA.

The tested cell lines displayed higher mitochondrial DNA content and a marked increase in mitochondrial respiration compared with primary ccRCC cells. These findings infer an elevated capacity to utilize other metabolic pathways in established cell lines, a lower dependence on glycolysis for energy production and thus a higher tolerance to glycolytic inhibition. Of note, ccRCC cells isolated from one patient did not respond to 3BrPA treatment (R104T). Seahorse analysis revealed a higher basal OCR and increased maximal respiratory capacity of these cells, suggesting a higher mitochondrial content and lower dependence on glycolysis for energy production. Together, these data imply that cells relying on functional glycolysis for energy production will be selectively sensitive to 3BrPA treatment.

The extreme glycolytic profile of primary ccRCC cells is probably a property of tumor cells *in vivo*, retained in primary ccRCC cells, that is lost in cell lines during establishment and propagation. These results point to the importance of using relevant model systems, and that established cell lines not always give a correct reflection of the tumor type they are derived from. Hence, screening for metabolic inhibitors using established cell lines might give misleading results and promising candidates might be overlooked.

In summary, our results demonstrate that primary ccRCC tumor cells are highly sensitive to glycolytic inhibition by 3BrPA, suggesting that the possibility to utilize pharmaceuticals targeting glycolysis, thereby disrupting energy metabolism, should be further studied in ccRCC tumors.

## Materials and Methods

### Cell culture and isolation of primary cells

Primary human ccRCC cells were isolated and cultured from patient nephrectomies as described previously,^30^ with ethical approval from the Lund University ethical committee (LU680-08 and LU 289-07). Tumors were classified as ccRCCs by an experienced pathologist. Normal samples were collected from healthy kidney cortex farthest from the tumor. 786-O cells were obtained from ATCC. SKRC10 and SKRC17 were kindly provided by E. Oosterwijk (Radboud University Nijmegen Medical Center, Nijmegen, The Netherlands). All cells were grown in DMEM high glucose supplemented with 10% FBS and 1% penicillin/streptavidin (Thermo Scientific, Waltham, MA, USA), except for SKRC7, SKRC10, SKRC17, SKRC21 and SKRC52, which were kept in RPMI. All cell lines were regularly checked for mycoplasma.

### 3-Bromopyruvate

3-Bromopyruvate was obtained from Sigma-Aldrich (St. Louis, MO, USA). A fresh stock solution of 20 mM 3BrPA in PBS was prepared for each experiment.

### WST-1 cell growth assay

Cells were seeded in triplicates in 96-well plates. The following day 3BrPA or vehicle was added in a total volume of 100 *μ*l media and cells were incubated for 1–6 days. WST-1 (10 *μ*l) solution (Roche, Basel, Switzerland) was added to cells and absorbance was measured after 4 h incubation.

### Nucleocounter cell viability assay

Cells were seeded in 24-well plates and incubated with or without 3BrPA for 24 h. Adherent and floating cells were collected and the number of live and dead cells was determined using the cell count and viability assay on the Nucleocounter NC3000 (ChemoMetec, Allerod, Denmark) according to the manufacturer's protocol.

### Measurement of mitochondrial respiration

Measurement of OCR was performed on a Seahorse XF24 Extracellular Flux Analyzer (SeaHorse Bioscience, North Billerica, MA, USA). Cells were seeded to confluency in a XF24 Seahorse assay plate. The following day media was changed to unbuffered XF Assay medium and the plate was incubated in a non-CO_2_ chamber for 2 h after which the OCR was analyzed according to the manufacturer's instructions. Maximal respiratory capacity was determined after injection of 4 *μ*g/ml oligomycin (Sigma-Aldrich), 4 *μ*M FCCP (Abcam, Cambridge, UK) and 5 *μ*M Rotenone (Sigma-Aldrich) according to instructions for the Cell Mito Stress Test. OCR was corrected to number of cells. Each sample was run in three to five replicates.

### Electron microscopy

Tissue samples were fixed in 2% paraformaldehyde and 1% glutaraldehyde in 0.1 M Sorensen phosphate buffer PH 7.4, followed by 1% Osmiumtetroxid in 0.5 M potassium cyanoferrate buffer. After dehydration, embedding and sectioning, samples were analyzed at the Lund University Bioimaging Center using a Tecnai biotwin 120 kV transmission electron microscope (FEI, Hillsboro, OR, USA).

### Mitochondrial DNA content

Total DNA was isolated using the Allprep RNA/DNA kit from Qiagen (Limburg, Netherlands). DNA samples were diluted to 0.4 ng/*μ*l and a quantitative PCR was performed using primers for nuclear and mitochondrial encoded genes. The relative mitochondrial DNA content of each sample was determined by dividing the levels of mitochondrial DNA by the nuclear encoded genes. For primer sequences see Supplementary Table 2.

### Lactate assay

Cells were seeded into 96-well plates. The following day, 3BrPA was added to the media and 1 h later lactate concentration in cell medium was determined spectrophotometrically using a Lactate Assay Kit from BioVision (Milpitas, CA, USA) according to the manufacturer's instructions. The assay was performed under serum-free conditions.

### Intracellular ATP measurement

Intracellular ATP levels were determined using the Cell Viability Kit SL (BioThema, Handen, Sweden). Cells were seeded in triplicates in a 96-well plate. Twenty-four hours later cells were treated with 3BrPA for 1 h, after which intracellular ATP levels were determined according to the manufacturer's instructions.

### Immunohistochemistry

Tissue samples or cultured cells were fixed in 4% neutral buffered formalin and embedded in paraffin according to the standard protocols. Immunohistochemical stainings were performed as described previously.^30^ Antibodies used were anti-MTCO2 (Abcam), anti-SLC16A1 (Sigma-Aldrich) and anti-Glut1 (Chemicon, Merck Millipore, Darmstadt, Germany).

### Quantitative Real-Time PCR

Total RNA was isolated using the the RNeasy mini kit from Qiagen. cDNA synthesis was performed using M-MuLV reverse transcriptase and random hexamers (Thermo Scientific). Quantitative real-time PCR was performed with the SYBR Green PCR Master Mix (Thermo Scientific) on an Mx 3005 P QPCR system (Agilent Technologies, Santa Clara, CA, USA). The comparative *C*_t_ method was used to quantify relative RNA levels and three separate housekeeping genes (HMBS, RPL13A, YWHAZ) were used for normalization. Primer sequences are listed in Supplementary Table 2.

### FACS analysis

Flow cytometry was performed on a FACS Calibur (BD Biosciences, San Jose, CA, USA). Percentage of apoptotic double-positive cells was determined after staining with Propidium Iodide (Sigma-Aldrich) and Annexin V-APC (BD Biosciences) according to the manufacturer's protocols. Cell cycle analysis was performed on fixated cells stained with Propidium Iodide. The percentage of cells in each stage of the cell cycle was determined using the Dean/Jett/Fox algorithm in the FlowJo software (Tree Star Inc., Ashland, OR, USA).

### Bioinformatic analysis

Level 3 RNA-seq data containing mRNA gene-level RSEM estimates were downloaded from The Cancer Genome Atlas (TCGA) data portal (http://tcga-data.nci.nih.gov/tcga/dataAccessMatrix.htm) by November 2013. The data comprised 505 ccRCCs and 70 normal kidney specimens analyzed on the Illumina HiSeq platform. RNA-seq data from the following cancer types included in the TCGA data collection were also downloaded for comparisons: BLCA, Bladder Urothelial Carcinoma; BRCA, Breast invasive carcinoma; COAD, Colon adenocarcinoma; GBM, Glioblastoma multiforme; HNSC, Head and Neck squamous cell carcinoma; KICH, Kidney Chromophobe; KIRC, Kidney renal clear cell carcinoma; LAML, Acute Myeloid Leukemia; LGG, Brain Lower Grade Glioma; LUAD, Lung adenocarcinoma; LUSC, Lung squamous cell carcinoma; OV, Serous Cystadenocarcinoma; PRAD, Prostate adenocarcinoma; READ, Rectum adenocarcinoma; SKCM, Stomach adenocarcinoma; THCA, Thyroid Carcinoma; UCEC, Uterine Corpus Endometrial Carcinoma. RSEM estimate values were multiplied by 10^6^ followed by adding an offset of 10 and subsequently log2 transformed. Two additional data sets (Wang *et al.* GSE14762^31^ and Liu *et al.* GSE16441^32^) with mRNA expression data of ccRCC and normal kidney were obtained from the Gene Set Omnibus data repository (GEO; http://www.ncbi.nlm.nih.gov/geo/).

## Abbreviations

ccRCC: clear cell renal cell carcinoma
pVHL: von Hippel Lindau protein
3BrPA: 3-bromopyruvate
HIF-1*α*: hypoxia inducible factor 1α
HIF-2α: hypoxia inducible factor 2α
TCGA: The Cancer Genome Atlas
MTCO2: mitochondrial encoded cytochrome c oxidase II
OCR: oxygen consumption rate
MCT1: monocarboxylate transporter 1

## References

[bib1] Ferlay J, Soerjomataram I, Ervik M, Dikshit R, Eser S, Mathers C, Rebelo M et al. Cancer Incidence and Mortality Worldwide: IARC CancerBase No. 11. 2013. Available from: http://globocan.iarc.fr. 10.1002/ijc.29210 25220842

[bib2] Moch H. An overview of renal cell cancer: pathology and genetics. Semin Cancer Biol 2013; 23: 3–9. 22722066 10.1016/j.semcancer.2012.06.006

[bib3] Canda AE, Kirkali Z. Current management of renal cell carcinoma and targeted therapy. Urol J 2006; 3: 1–14. 17590846

[bib4] Valera VA, Merino MJ. Misdiagnosis of clear cell renal cell carcinoma. Nat Rev Urol 2011; 8: 321–333. 21587224 10.1038/nrurol.2011.64

[bib5] The Cancer Genome Atlas Research. Comprehensive molecular characterization of clear cell renal cell carcinoma. Nature 2013; 499: 43–49. 23792563 10.1038/nature12222 PMC3771322

[bib6] Zbar B, Brauch H, Talmadge C, Linehan M. Loss of alleles of loci on the short arm of chromosome 3 in renal cell carcinoma. Nature 1987; 327: 721–724. 2885753 10.1038/327721a0

[bib7] Semenza GL. Hypoxia-inducible factors in physiology and medicine. Cell 2012; 148: 399–408. 22304911 10.1016/j.cell.2012.01.021 PMC3437543

[bib8] Kaelin WG Jr. The von Hippel-Lindau tumor suppressor protein and clear cell renal carcinoma. Clin Cancer Res 2007; 13: 680 s–684s. 10.1158/1078-0432.CCR-06-1865 17255293

[bib9] Ward PS, Thompson CB. Metabolic reprogramming: a cancer hallmark even warburg did not anticipate. Cancer Cell 2012; 21: 297–308. 22439925 10.1016/j.ccr.2012.02.014 PMC3311998

[bib10] Gatto F, Nookaew I, Nielsen J. Chromosome 3p loss of heterozygosity is associated with a unique metabolic network in clear cell renal carcinoma. Proc Natl Acad Sci USA 2014; 111: E866–E875. 24550497 10.1073/pnas.1319196111 PMC3948310

[bib11] Unwin RD, Craven RA, Harnden P, Hanrahan S, Totty N, Knowles M et al. Proteomic changes in renal cancer and co-ordinate demonstration of both the glycolytic and mitochondrial aspects of the Warburg effect. Proteomics 2003; 3: 1620–1632. 12923786 10.1002/pmic.200300464

[bib12] Perroud B, Lee J, Valkova N, Dhirapong A, Lin PY, Fiehn O et al. Pathway analysis of kidney cancer using proteomics and metabolic profiling. Mol Cancer 2006; 5: 64. 17123452 10.1186/1476-4598-5-64 PMC1665458

[bib13] Meierhofer D, Mayr JA, Foetschl U, Berger A, Fink K, Schmeller N et al. Decrease of mitochondrial DNA content and energy metabolism in renal cell carcinoma. Carcinogenesis 2004; 25 p 1005–1010. 14764459 10.1093/carcin/bgh104

[bib14] Liu J, Li M, Song B, Jia C, Zhang L, Bai X et al. Metformin inhibits renal cell carcinoma *in vitro* and *in vivo* xenograft. Urol Oncol 2013; 31: 264–270. 21676631 10.1016/j.urolonc.2011.01.003

[bib15] Nangia-Makker P, Yu Y, Vasudevan A, Farhana L, Rajendra SG, Levi E et al. Metformin: a potential therapeutic agent for recurrent colon cancer. PLoS ONE 2014; 9: e84369. 24465408 10.1371/journal.pone.0084369 PMC3896365

[bib16] Hadad SM, Hardie DG, Appleyard V, Thompson AM. Effects of metformin on breast cancer cell proliferation, the AMPK pathway and the cell cycle. Clin Transl Oncol 2013; 16: 746–752. 24338509 10.1007/s12094-013-1144-8

[bib17] Ko YH, Pedersen PL, Geschwind JF. Glucose catabolism in the rabbit VX2 tumor model for liver cancer: characterization and targeting hexokinase. Cancer Lett 2001; 173: 83–91. 11578813 10.1016/s0304-3835(01)00667-x

[bib18] Dell'Antone P. Targets of 3-bromopyruvate, a new, energy depleting, anticancer agent. Med Chem 2009; 5: 491–496. 19534685 10.2174/157340609790170551

[bib19] Sjolund J, Johansson M, Manna S, Norin C, Pietras A, Beckman S et al. Suppression of renal cell carcinoma growth by inhibition of Notch signaling *in vitro* and *in vivo*. J Clin Invest 2008; 118: 217–228. 18079963 10.1172/JCI32086 PMC2129233

[bib20] Birsoy K, Wang T, Possemato R, Yilmaz OH, Koch CE, Chen WW et al. MCT1-mediated transport of a toxic molecule is an effective strategy for targeting glycolytic tumors. Nat Genet 2013; 45: 104–108. 23202129 10.1038/ng.2471 PMC3530647

[bib21] Semenza GL. HIF-1 mediates the Warburg effect in clear cell renal carcinoma. J Bioenerg Biomembr 2007; 39: 231–234. 17551816 10.1007/s10863-007-9081-2

[bib22] Solenski NJ, diPierro CG, Trimmer PA, Kwan AL, Helm GA. Ultrastructural changes of neuronal mitochondria after transient and permanent cerebral ischemia. Stroke 2002; 33: 816–824. 11872909 10.1161/hs0302.104541

[bib23] Posadas EM, Limvorasak S, Sharma S, Figlin RA. Targeting angiogenesis in renal cell carcinoma. Expert Opin Pharmacother 2013; 14: 2221–2236. 23984807 10.1517/14656566.2013.832202

[bib24] Ko YH, Smith BL, Wang Y, Pomper MG, Rini DA, Torbenson MS et al. Advanced cancers: eradication in all cases using 3-bromopyruvate therapy to deplete ATP. Biochem Biophys Res Commun 2004; 324: 269–275. 15465013 10.1016/j.bbrc.2004.09.047

[bib25] Pedersen PL. 3-Bromopyruvate (3BP) a fast acting, promising, powerful, specific, and effective "small molecule" anti-cancer agent taken from labside to bedside: introduction to a special issue. J Bioenerg Biomembr 2012; 44: 1–6. 22382780 10.1007/s10863-012-9425-4

[bib26] Yoshino H, Enokida H, Itesako T, Kojima S, Kinoshita T, Tatarano S et al. Tumor-suppressive microRNA-143/145 cluster targets hexokinase-2 in renal cell carcinoma. Cancer Sci 2013; 104: 1567–1574. 24033605 10.1111/cas.12280 PMC7653528

[bib27] Rodrigues-Ferreira C, da Silva AP, Galina A. Effect of the antitumoral alkylating agent 3-bromopyruvate on mitochondrial respiration: role of mitochondrially bound hexokinase. J Bioenerg Biomembr 2012; 44: 39–49. 22322891 10.1007/s10863-012-9413-8

[bib28] Pereira da Silva AP, El-Bacha T, Kyaw N, dos Santos RS, da-Silva WS, Almeida FC et al. Inhibition of energy-producing pathways of HepG2 cells by 3-bromopyruvate. Biochem J 2009; 417: 717–726. 18945211 10.1042/BJ20080805

[bib29] Sanborn BM, Felberg NT, Hollocher TC. The inactivation of succinate dehydrogenase by bromopyruvate. Biochim Biophys Acta 1971; 227: 219–231. 5550821 10.1016/0005-2744(71)90055-6

[bib30] Bostrom AK, Möller C, Nilsson E, Elfving P, Axelson H, Johansson ME. Sarcomatoid conversion of clear cell renal cell carcinoma in relation to epithelial-to-mesenchymal transition. Hum Pathol 2012; 43: 708–719. 21992819 10.1016/j.humpath.2011.06.019

[bib31] Wang Y, Roche O, Yan MS, Finak G, Evans AJ, Metcalf JL et al. Regulation of endocytosis via the oxygen-sensing pathway. Nat Med 2009; 15: 319–324. 19252501 10.1038/nm.1922

[bib32] Liu H, Brannon AR, Reddy AR, Alexe G, Seiler MW, Arreola A, Oza JH et al. Identifying mRNA targets of microRNA dysregulated in cancer: with application to clear cell renal cell carcinoma. BMC Syst Biol 2010; 4: 51. 20420713 10.1186/1752-0509-4-51 PMC2876063

