# Supplementary figures and images for: Primary clear cell renal carcinoma cells display minimal mitochondrial respiratory capacity resulting in pronounced sensitivity to glycolytic inhibition by 3-Bromopyruvate
Source: Cell Death Dis. 2015 Jan 8;6(1):e1585–. doi: 10.1038/cddis.2014.545 (PMC4669744; doi:10.1038/cddis.2014.545)

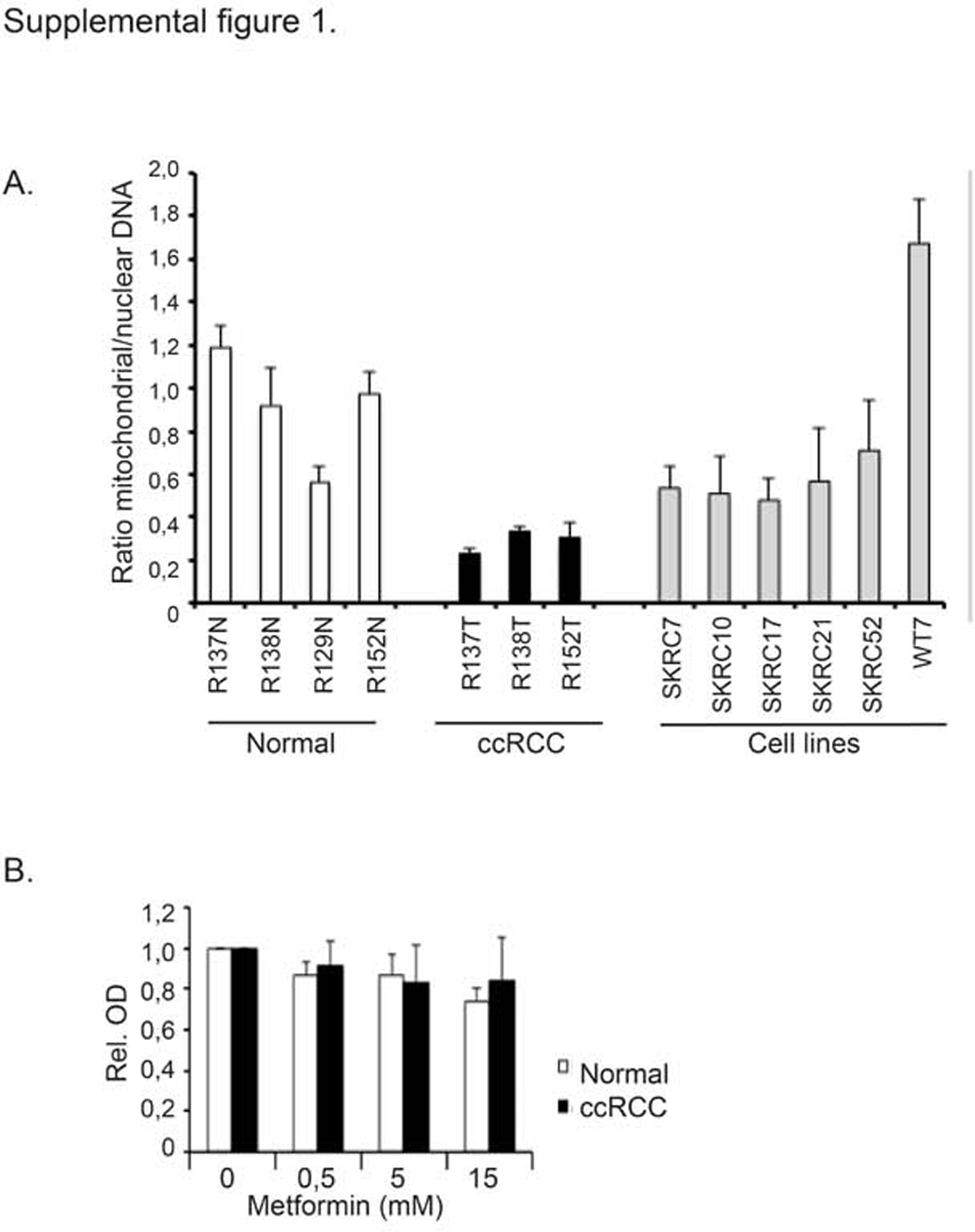

Supplement: Supplementary Figure 1 [file cddis2014545x1.tif]

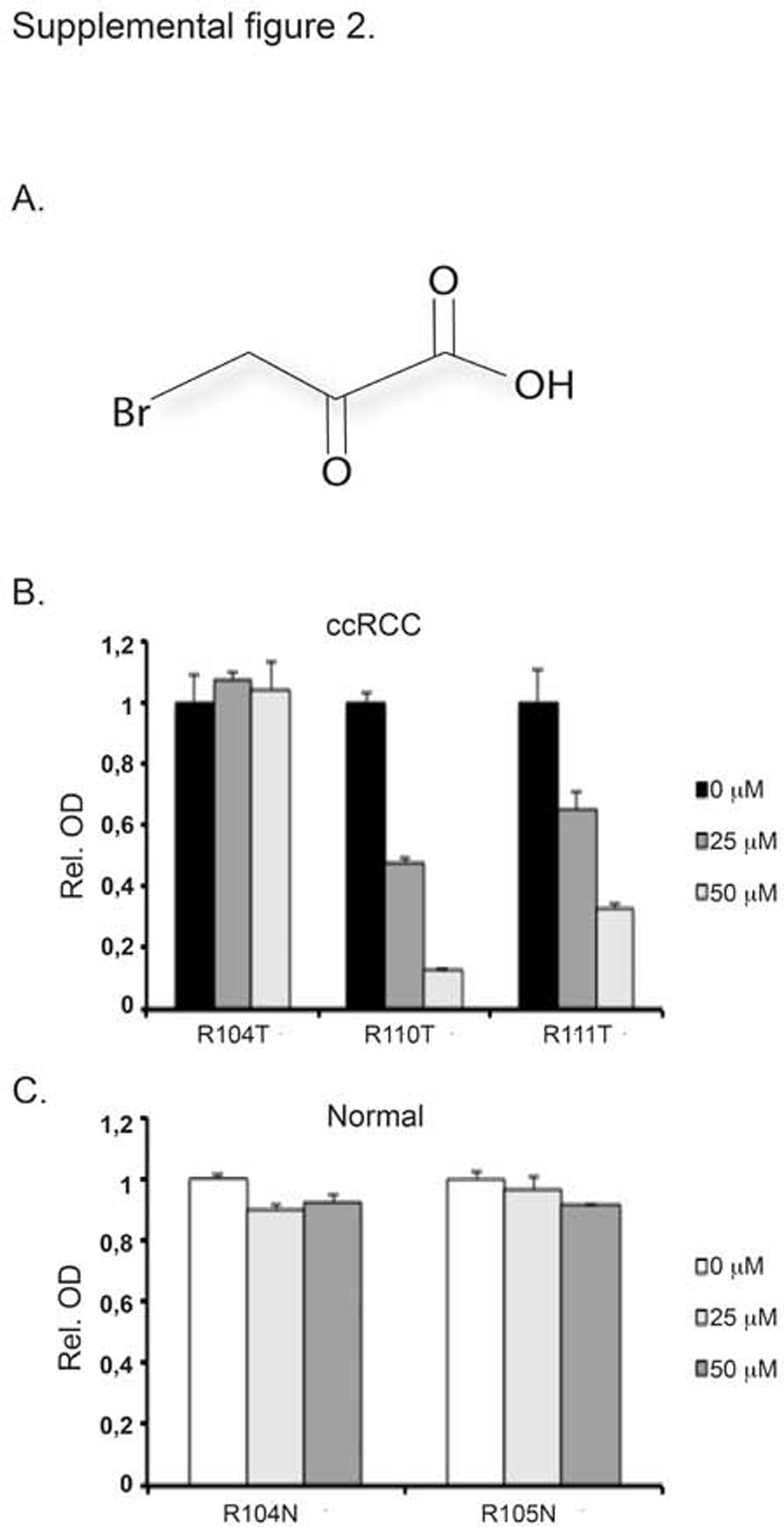

Supplement: Supplementary Figure 2 [file cddis2014545x2.tif]

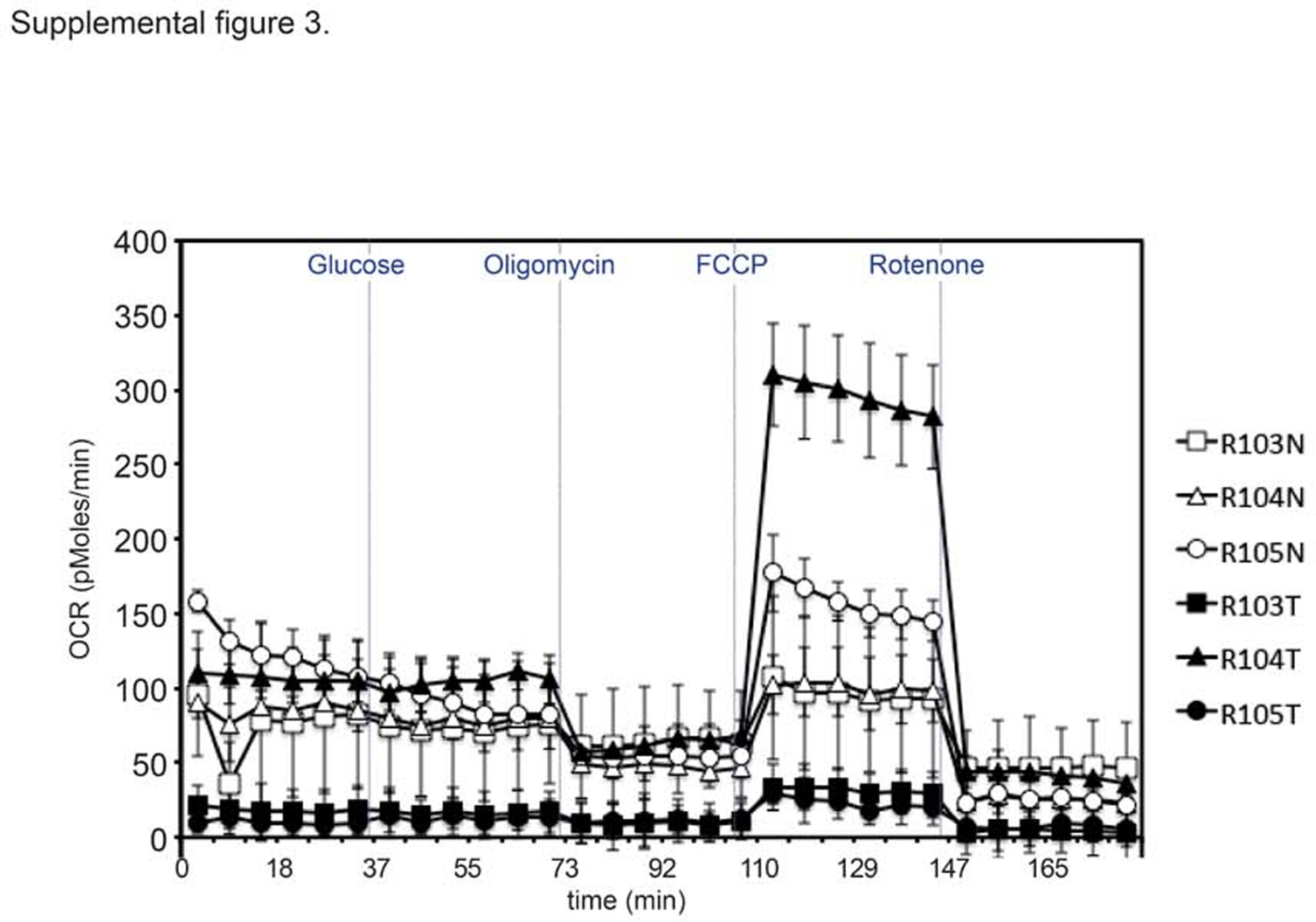

Supplement: Supplementary Figure 3 [file cddis2014545x3.tif]

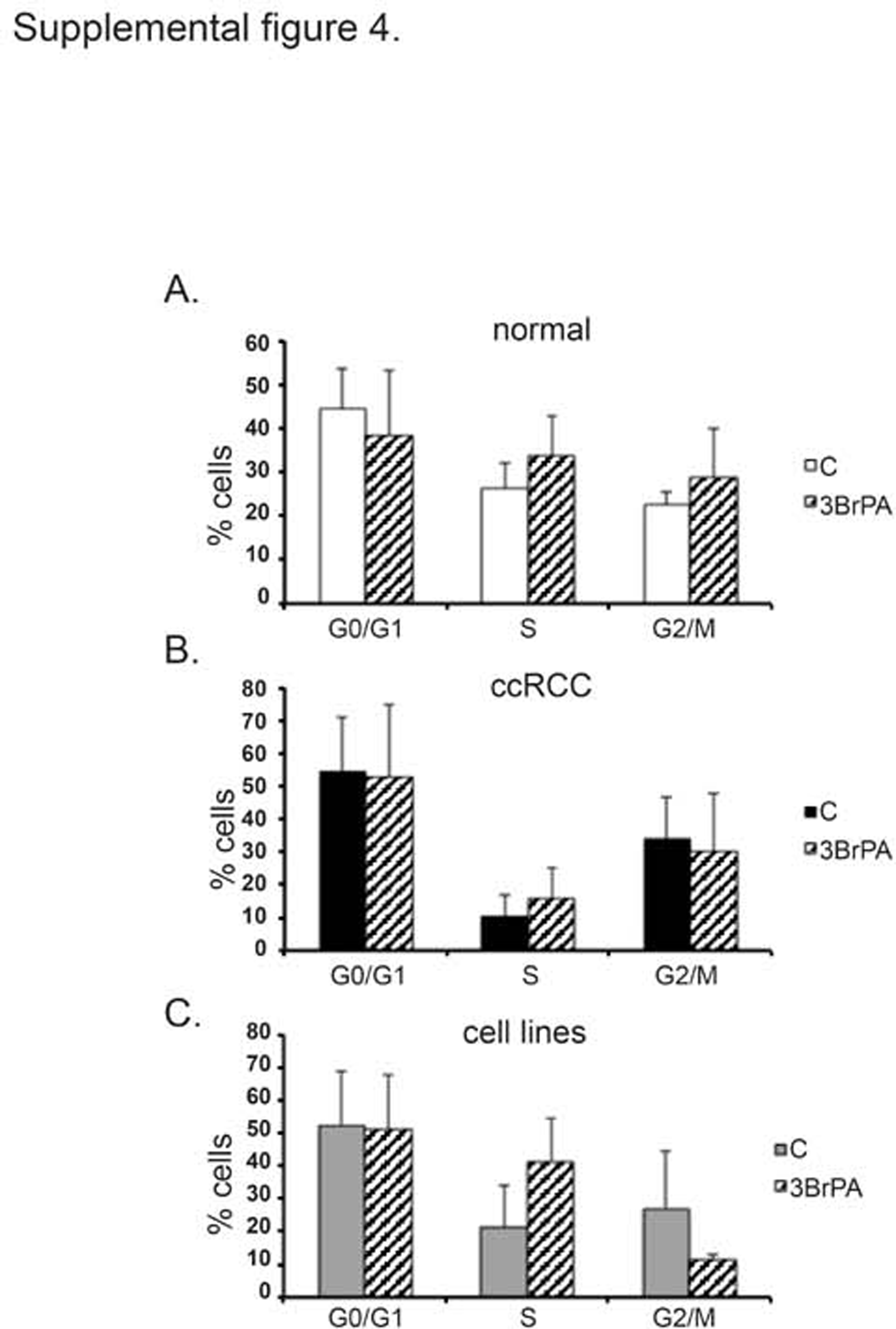

Supplement: Supplementary Figure 4 [file cddis2014545x4.tif]
