# Supplementary material for: Primary clear cell renal carcinoma cells display minimal mitochondrial respiratory capacity resulting in pronounced sensitivity to glycolytic inhibition by 3-Bromopyruvate
Source: Cell Death Dis. 2015 Jan 8;6(1):e1585–. doi: 10.1038/cddis.2014.545 (PMC4669744; doi:10.1038/cddis.2014.545)
Supplement: Supplementary Figure Legends [file cddis2014545x5.doc]

**SUPPLEMENTAL FIGURE LEGENDS**

Supplemental Figure 1.

A. Ratio between mitochondrial and nuclear DNA content in primary normal and ccRCC cells, as well as established cell lines, determined by quantitative PCR.

B. Primary ccRCC cells are insensitive to Metformin treatment. WST-1 assay showing the effect on primary cultures of treatment with the complex I-inhibitor Metformin for 24 hours. Data shown are a summary from three different normal (R105N, R156N, R117N) and ccRCC samples (R105T, R156T, R157T).

Supplemental Figure 2.

Structure of 3BrPA (A). WST-1 assay showing the effect of treatment with 3BrPA for six days in primary ccRCC (B) or normal (C) cells.

Supplemental Figure 3.

Results from the Seahorse XF24 analyzer showing OCR levels in three sets of normal primary and ccRCC cells (R103 N/T, R104 N/T and R105 N/T). Note that the ccRCC culture R104T display a markedly higher basal OCR compared to the other tumor cells, and even an increased respiratory capacity as compared to the normal cells. Data are normalized to the number of cells.

Supplemental Figure 4.

Cell cycle distribution in primary normal (A), ccRCC (B) and established cell lines (C) after 24 hours treatment with 50 μM 3BrPA.
