# Supplementary material for: Primary clear cell renal carcinoma cells display minimal mitochondrial respiratory capacity resulting in pronounced sensitivity to glycolytic inhibition by 3-Bromopyruvate
Source: Cell Death Dis. 2015 Jan 8;6(1):e1585–. doi: 10.1038/cddis.2014.545 (PMC4669744; doi:10.1038/cddis.2014.545)
Supplement: Supplementary Table 1 [file cddis2014545x6.doc]

Supplemental Table 1. IC_50_ values describing the sensitivity of the indicated cultures to 3BrPA treatment.

|  |  | ***IC_50_(μM)*** |
| --- | --- | --- |
| Normal | R132N | 164,4 |
|  | R135N | 187,1 |
|  | R137N | 143,8 |
|  | R138N | 143,3 |
|  |  |  |
| ccRCC | R132T | 49,2 |
|  | R122T | 50,8 |
|  | R138T | 89,4 |
|  | R143T | 54,8 |
|  |  |  |
| Cell lines | 786-O | 126,5 |
|  | WT-7 | 120,3 |
|  | SKRC10 | 93,7 |
|  | SKRC17 | 98,0 |
|  | SKRC7 | 101,4 |
|  | SKRC21 | 112,1 |
|  | SKRC52 | 90,6 |
