# Supplementary material for: Primary clear cell renal carcinoma cells display minimal mitochondrial respiratory capacity resulting in pronounced sensitivity to glycolytic inhibition by 3-Bromopyruvate
Source: Cell Death Dis. 2015 Jan 8;6(1):e1585–. doi: 10.1038/cddis.2014.545 (PMC4669744; doi:10.1038/cddis.2014.545)
Supplement: Supplementary Table 2 [file cddis2014545x7.doc]

Supplemental Table 2. Primer sequences.

| ***Target gene*** | ***Forward sequence 5’-3’*** | ***Reverse sequence 5’-3’*** |
| --- | --- | --- |
| mt3212 | CACCCAAGAACAGGGTTTGT | TGGGCCATCCCTATGTTGTTAA |
| 18S | TAGAGGGACAAGTGGCGTTC | CGCTGAGCCAGTCAGTGT |
| H4c | GGGATAACATCCAGGGCATT | CCCTGACGTTTTAGGGCATA |
| mitNT7773 | CCGTCTGAACTATCCTGCCC | GCCGTAGTCGGTGTACTCGT |
| HMBS | GGCAATGCGGCTGCAA | GGGTACCCACGCGAATCAC |
| RPL13A | CCTGGAGGAGAAGAGGAAAGAGA | TTGAGGACCTCTGTGTATTTGTCAA |
| YWHAZ | ACTTTTGGTACATTGTGGCTTCAA | CCGCCAGGACAAACCAGTAT |
| SLC16A1 | TTATAAGGCAGCCTCGCTGG | AGCTCCAATTACCACTGCCC |
